# *Burkholderia* Gut Symbionts Associated with European and Japanese Populations of the Dock Bug *Coreus marginatus* (Coreoidea: Coreidae)

**DOI:** 10.1264/jsme2.ME19011

**Published:** 2019-06-06

**Authors:** Tsubasa Ohbayashi, Hideomi Itoh, Joy Lachat, Yoshitomo Kikuchi, Peter Mergaert

**Affiliations:** 1 Institute for Integrative Biology of the Cell, UMR9198, CNRS, Université Paris-Sud, CEA Gif-sur-Yvette France; 2 Bioproduction Research Institute, National Institute of Advanced Industrial Science and Technology (AIST), Hokkaido Center 2–17–2–1 Tsukisamu-higashi, Toyohira-ku, Sapporo 062–8517 Japan; 3 Computational Bio Big Data Open Innovation Laboratory (CBBDOIL), AIST, Hokkaido Center 2–17–2–1 Tsukisamu-higashi, Toyohira-ku, Sapporo 062–8517 Japan

**Keywords:** *Burkholderia*, stinkbug, obligate gut symbiosis, region-dependent symbionts

## Abstract

Insects of the heteropteran superfamilies Coreoidea and Lygaeoidea are consistently associated with symbionts of a specific group of the genus *Burkholderia*, called the “stinkbug-associated beneficial and environmental (SBE)” group. The symbiosis is maintained by the environmental transmission of symbionts. We investigated European and Japanese populations of the dock bug *Coreus marginatus* (Coreoidea: Coreidae). High nymphal mortality in reared aposymbiotic insects suggested an obligate host-symbiont association in this species. Molecular phylogenetic analyses based on 16S rRNA gene sequences revealed that all 173 individuals investigated were colonized by *Burkholderia*, which were further assigned to different subgroups of the SBE in a region-dependent pattern.

The suborder Heteroptera is a diverse taxonomic group in insects and consists of 42,300 described species (9). Phytophagous members commonly possess symbiotic bacteria inside their bodies (1, 15, 19). While some heteropteran species harbor symbionts intracellularly (10, 20–22, 24), the majority of phytophagous species possess symbiotic bacteria extracellularly in the lumen of sac-like tissues, called “crypts”, in the posterior midgut (2, 8, 25). Members of the superfamily Pentatomoidea harbor specific bacterial symbionts belonging to *Gammaproteobacteria* (19). These symbionts are essential for host growth and reproduction and are vertically transmitted from mother to offspring. In contrast, most members of the superfamilies Lygaeoidea and Coreoidea are associated with betaproteobacterial symbionts of a specific clade in the genus *Burkholderia*, called the “stinkbug-associated beneficial and environmental (SBE)” group (7, 16, 30). The coreoid and lygaeoid species not vertically transmit *Burkholderia* symbionts, but they acquire them from environmental soil every generation (14, 17). At this stage, the biological effects of the *Burkholderia* symbiont have only been reported in the bean bug *Riptortus pedestris* (superfamily Coreoidea: family Alydidae), in which the symbiont is not essential, but significantly enhances the growth rate, body size, and fecundity of the bean bug host (14, 18).

We previously investigated 22 species of Coreoidea and Lygaeoidea, all of which were collected in Japan and harbored the SBE group *Burkholderia* (13, 16). Six species of American Coreoidea and Lygaeoidea were also examined and the symbiotic organs of these species were also dominated by SBE-group *Burkholderia* (1, 7, 26), although other groups of *Burkholderia* were also detected in some cases (1). A recent study on European and Japanese species of the spurge bug, *Dicranocephalus* spp. (superfamily Coreoidea: family Stenocephalidae), revealed that while the Japanese species are consistently associated with the SBE group *Burkholderia*, European species are more likely to harbor a distinct lineage of *Burkholderia*, tentatively named “Stenocephalidae-clade” *Burkholderia* (23). This finding suggests the geographical divergence of the stinkbug-*Burkholderia* association. However, it currently remains unclear whether the case of the spurge bug is exceptional.

The dock bug *Coreus marginatus* (superfamily Coreoidea: family Coreidae) (Fig. 1A) is broadly distributed in the Northern Hemisphere, from Europe over central Asia to Japan (11, 12). It feeds on the leaves and seeds of *Rumex* plants (Fig. 1B), and is a serious pest of *Rumex* herbs, such as sorrel (11). In the present study, we investigated the symbiotic bacteria of *C. marginatus*, which belong to the SBE, and examined their fitness effects on the host insect. We further clarified whether a region-dependent divergence of symbionts exists between European and Japanese host populations.

The dock bug possessed numerous crypts in the posterior region of the midgut. These crypts were white and arranged in two rows (Fig. 1C). To investigate the prevalence of *Burkholderia* in this species, wild populations collected in diverse locations of Europe and Japan were assessed by diagnostic PCR with a *Burkholderia*-specific primer set (29). The insects examined in the present study are listed in Table S1. The crypt region was dissected out by forceps under a binocular, and the symbiotic organ (M4 in Fig. 1C) was subjected to DNA extraction and diagnostic PCR as previously described (16). A total of 163 individuals from 16 European populations and ten individuals from two Japanese populations were investigated, all of which were positive for *Burkholderia* (Table S1). In contrast, no *Burkholderia* infection was observed in the egg samples of reared insects (positive/total tested=0/8), strongly suggesting that *C. marginatus* does not transmit the symbiont vertically, but acquires it from the environment, similar to other coreoid stinkbugs.

The *Burkholderia* symbiont of the dock bug was successfully isolated from the midgut crypts of an insect collected in Crèche Belle-Image, the CNRS campus, Gif-sur-Yvette, France on 24^th^ May 2017 by culturing the crypt content on a YG (yeast-glucose) agar plate, as previously described (16). A green fluorescence protein (GFP)-expressing derivative, constructed from this isolate as previously described (18) and fed to second instar nymphs that descended from wild insects collected at the same location (Crèche Belle-Image, CNRS-campus, Gif-sur-Yvette, France in 2017), showed a specific localization in the midgut crypts (Fig. 1D and E), confirming the gut symbiotic association between *Burkholderia* and the dock bug. Using this cultured strain, the fitness effects of the *Burkholderia* symbiont were investigated. Second instar nymphs were fed cultured *Burkholderia* 6 d after hatching and maintained in a clean plastic cup at 25°C under a long day regimen (16 h light, 8 h dark) by feeding on roasted pistachio and peanut seeds (*Pistacia vera* and *Arachis hypogaea*, respectively) and distilled water containing 0.05% ascorbic acid. While uninfected insects showed a survival rate of only 7.7% (survived/total=1/13), insect survival significantly improved to 52.5% (21/40) in infected insects (Fig. 1F), strongly suggesting an obligate host-symbiont relationship in the dock bug. In the case of the bean bug *R. pedestris*, the *Burkholderia* association is facultative: the symbiont does not strongly affect host survival, but does influence the growth and fecundity of the insect host (14, 18). Although the biological function of the *Burkholderia* symbiont remains unclear, metabolic dependency on the symbiont appears to differ between stinkbug species that feed on different host plants.

To clarify the phylogenetic placement of *Burkholderia* symbionts associated with dock bugs, selected individuals from the European and Japanese populations were subjected to a clone library analysis of a 1.5-kb fragment of the bacterial 16S rRNA gene, as previously described (16). Ten and four insects representing ten European and two Japanese populations, respectively, were investigated (Table S1). A total of 110 clones were sequenced and subjected to a BLAST search. The top BLAST hits of all sequences were the 16S rRNA gene sequences of *Burkholderia* species. The 110 sequences were classified into five OTUs (Table S2 and S3) based on the UCLUST clustering method with a 99% sequence identity threshold in QIIME (3). These results indicated that (i) 11 and three individuals were infected with single and multiple *Burkholderia* OTUs, respectively, and (ii) OTU3 was the most frequently detected and present in all European individuals and two out of four Japanese specimens (Table S2). Although the clone library analysis demonstrated that the *Burkholderia* composition is simple in the dock bug, this result needs to be confirmed in a more comprehensive analysis using deep sequencing of the bacterial content in midgut crypts.

The genus *Burkholderia* is grouped into three phylogenetically and ecologically distinct clades (6, 32). The first clade consists of many human, animal, and plant pathogens, including *B. cepacia*, *B. pseudomallei*, and *B. mallei*, designated as the “*Burkholderia cepacia* complex (BCC)” group. The second clade includes a number of plant growth-promoting rhizobacteria and nodule-forming plant symbionts, assigned as the “plant-associated beneficial and environmental (PBE)” group, which was recently nominated as a novel genus “*Paraburkholderia*” (28). The third clade mainly consists of gut symbionts of the Coreoidea and Lygaeoidea stinkbugs, assigned as SBE or the “*Burkholderia glathei* clade (BGC)”, for which the novel genus “*Caballeronia*” has been proposed (5). A recent genome-based phylogenetic study strongly suggested that the *Caballeronia* genus is subdivided into at least two clades: a clade consisting of stinkbug symbionts and leaf-nodule symbionts, and a second clade consisting of *B. glathei*, *B. sordidicola*, and their allied species (31, 32). The former and latter clades are named here as “SBE Group α” and “SBE Group β”, respectively. Symbionts of the European spurge bugs are mostly grouped into SBE Group β (23) (Fig. 2).

The phylogenetic placement of the *Burkholderia* OTUs detected from the dock bug is shown in Fig. 2. OTU1 and OTU2, detected in two Japanese populations and one French population of the dock bug, were placed in SBE group α, in which OTUs were clustered with *Burkholderia* detected from Japanese and American coreoid and lygaeoid stinkbugs (Fig. 2, Table S2). The three other OTUs, including OTU3 detected in most European dock bug populations, were placed in SBE group β (Fig. 2). It is important to note that all of the ten insects investigated in seven European countries (France, Germany, Belgium, Italy, Hungary, Denmark, and Ukraine) were almost exclusively associated with *Burkholderia* of SBE group β (Table S2). Based on our previous findings on spurge bugs (23), it is plausible that coreoid stinkbugs inhabiting Europe are consistently associated with this specific clade of *Burkholderia*. Recent worldwide surveys revealed a “region-dependent pattern” of soil microbiota (4, 27), which may affect the region-dependent *Burkholderia* infection of stinkbugs. To clarify this point, further worldwide surveys on both soils and inhabiting stinkbugs are needed.

The nucleotide sequence data of the 16S rRNA gene obtained in the present study have been deposited in the DDBJ/EMBL/GenBank public databases with the accession numbers LC441114–LC441145 and LC455791–LC455869 (summarized in Table S1).

## Supplementary Information



## Figures and Tables

**Fig. 1:**
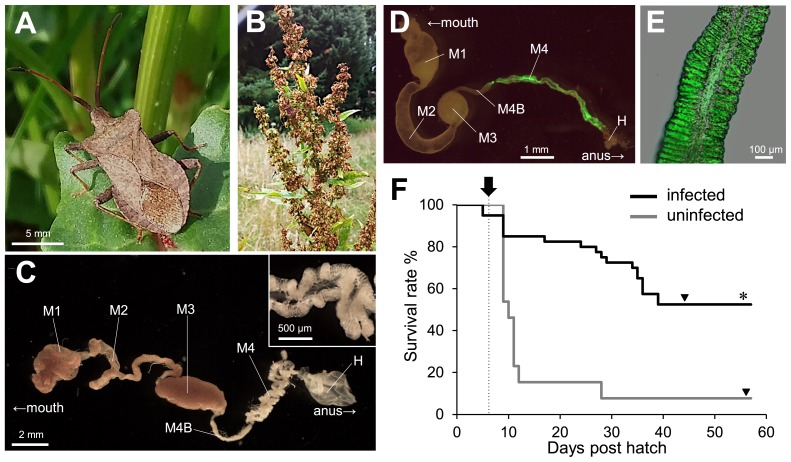
The dock bug *Coreus marginatus* and its gut symbiotic association. (A) An adult female of *C. marginatus*. (B) *Rumex* host plant. (C) A dissected midgut of an adult male. M1 midgut first section, M2 midgut second section, M3 midgut third section, M4B midgut fourth section with bulb, M4 midgut fourth section with crypts (symbiotic organ), H hindgut. The inset shows an enlarged image of crypt-bearing M4. (D) A dissected midgut of a 3^rd^ instar nymph infected with a GFP-labeled symbiont. (E) An enlarged image of crypts in M4 colonized by GFP-labeled *Burkholderia*. (F) Survival rate of *C. marginatus* infected with *Burkholderia* (black line, *n*=40) or uninfected (gray line, *n*=13). An inoculation was performed at 6 d post hatch (arrow with dotted line). Symbiotic insects (21 survivors) molted to adults at 44.2±4.0 d post hatch, and aposymbiotic ones (only 1 survivor) at 57 d post hatch (arrowheads). The survival rate of symbiotic insects was significantly higher than that of aposymbiotic ones (*P<0.01, Fisher’s exact test).

**Fig. 2:**
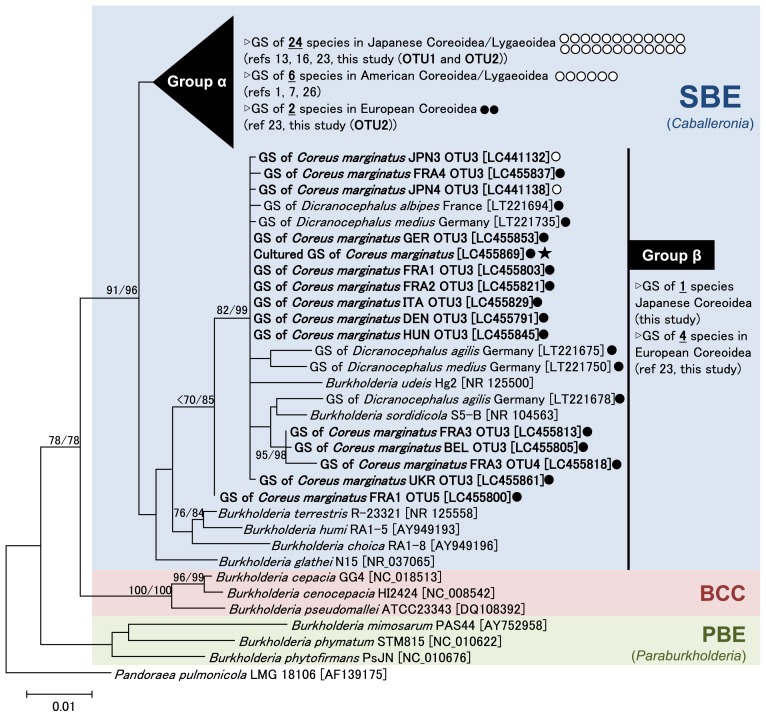
Molecular phylogeny of gut symbiotic *Burkholderia* of the dock bug shown by a neighbor-joining tree based on 1,332 aligned nucleotide sites of the 16S rRNA gene. The major *Burkholderia* clades (SBE, BCC, and PBE) as well as “SBE Group α” and “SBE Group β” are indicated. SBE Group α is a large group containing the gut symbionts of most Japanese and American species of the Coreoidea/Lygaeoidea (1, 7, 13, 16, 23, 26). An uncompressed tree of this group is shown in Fig. S1. SBE Group β was described as the “Stenocephalidae clade” in a previous study (23) and includes *B. glathei*, *B. sordidicola*, and most of the OTUs detected from European populations of the dock bug. Accession numbers in the DNA database (DDBJ/EMBL/GenBank) are shown in square brackets. Bootstrap values higher than 70% are indicated at the nodes in the order of maximum likelihood/neighbor-joining (1,000 replicates). Maximum likelihood phylogeny was estimated using the neighbor-joining tree as an initial guide tree. OTUs examined in the present study are shown in bold case. Closed circles: symbionts detected from European stinkbug populations. Open circles: symbionts detected from Japanese and American stinkbug populations. Asterisk: a cultured strain isolated from *C. marginatus* collected in Crèche Belle-Image, the CNRS campus, Gif-sur-Yvette, France. GS: gut symbiont.
