# Supplementary material for: Burkholderia Gut Symbionts Associated with European and Japanese Populations of the Dock Bug Coreus marginatus (Coreoidea: Coreidae)
Source: Microbes Environ. 2019 Jun 6;34(2):219–22. doi: 10.1264/jsme2.ME19011 (PMC6594735; doi:10.1264/jsme2.ME19011)
Supplement: Supplementary file 1 [file 34_219_s1.pdf]

# Fig. S1

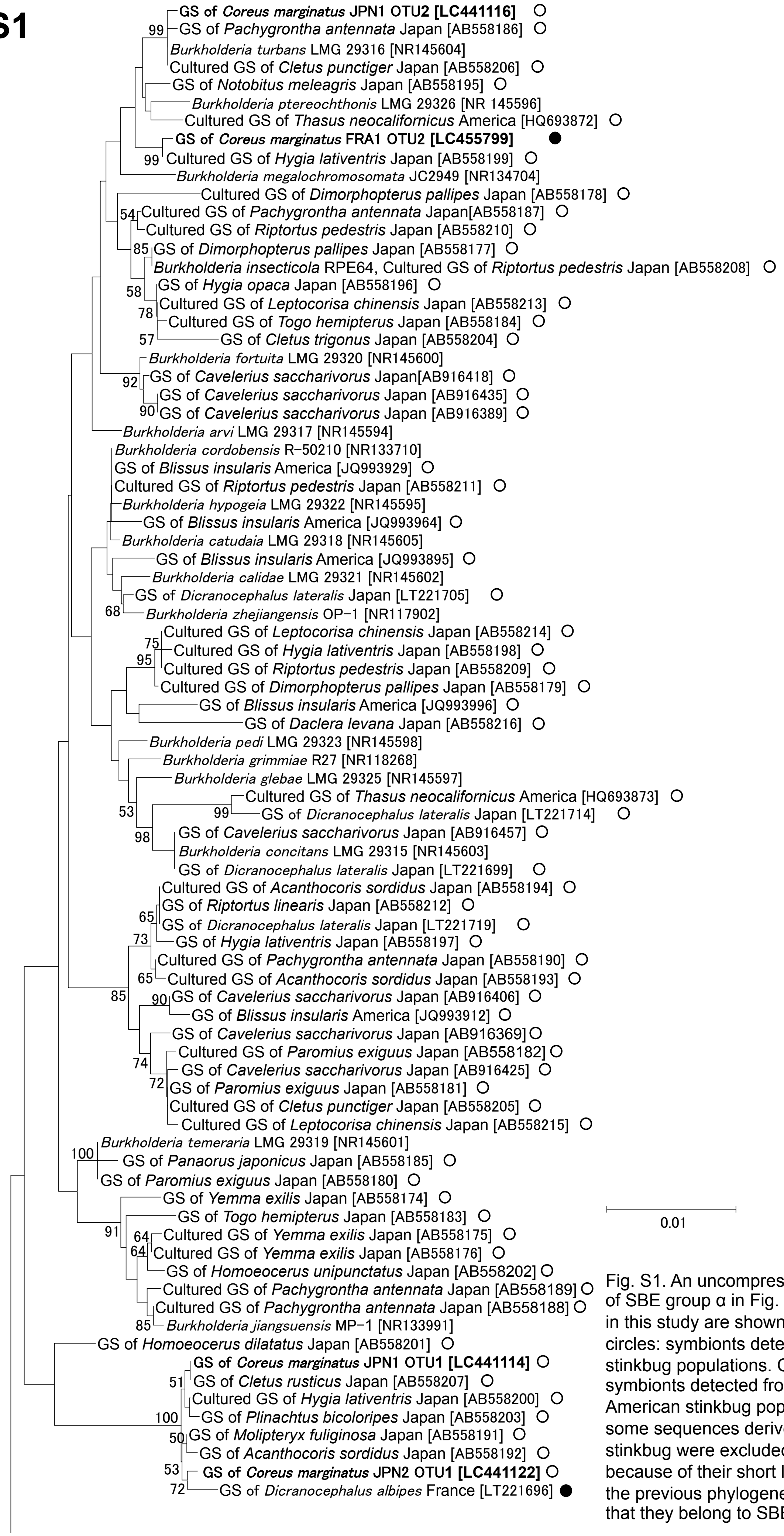


Fig. S1. An uncompressed phylogenetic tree of SBE group α in Fig. 2. OTUs determined in this study are shown in bold case. Closed circles: symbionts detected from European stinkbug populations. Open circles: symbionts detected from Japanese and American stinkbug populations. Although some sequences derived from American stinkbug were excluded from this tree because of their short length (<1,000 bp), the previous phylogenetic analysis revealed that they belong to SBE group α (ref. 7).

**Table S1.** Details of *Coreus marginatus* samples inspected in this study

| Collection site | | Instar | Collector | Collection Date | Diagnostic PCR[a] | Clone library analysis (Sample ID) | Accession number |
|---|---|---|---|---|---|---|---|
| France | Crèche Belle-Image, CNRS-campus, Gif-sur-Yvette | Adult | P. Mergaert, T. Ohbayashi | 30th May, 2017 | 100% (11/11) | - | - |
| | Bridge Mérantaise, CNRS-campus, Gif-sur-Yvette | Adult | P. Mergaert, T. Ohbayashi | 30th May, 2017 | 100% (5/5) | 6 clones from 1 insect (FRA1) | LC455799-LC455804 |
| | Building 21, CNRS-campus, Gif-sur-Yvette | Adult | P. Mergaert, T. Ohbayashi | 30th May, 2017 | 100% (12/12) | - | - |
| | Bassin south, Bures-sur-Yvette | Adult | P. Mergaert | 30th May, 2017 | 100% (11/11) | 8 clones from 1 insect (FRA2) | LC455821-LC455828 |
| | Paris-sud university campus, Bures-sur-Yvette[b] | Adult | P. Mergaert | 30th May, 2017 | 100% (7/7) | - | - |
| | Paris-sud university campus, Bures-sur-Yvette[b] | 3rd; 4th | P. Mergaert | 20th July, 2017 | 100% (11/11) | - | - |
| | Courtaboeuf, Orsay | Adult | P. Mergaert | 5th June , 2017 | 100% (10/10) | - | - |
| | Ferme Viltain, Jouy-en-Josas | Adult | P. Mergaert | 5th June , 2017 | 100% (2/2) | - | - |
| | Cancale | 3rd; 4th | P. Mergaert | 5th August, 2017 | 100% (12/12) | 8 clones from 1 insect (FRA3) | LC455813-LC455820 |
| | Rouen | Adult; 4th | P. Mergaert | 6th August, 2017 | 100% (9/9) | 8 clones from 1 insect (FRA4) | LC455837-LC455844 |
| Germany | Munich | Adult | P. Mergaert | 10th June, 2017 | 100% (4/4) | 8 clones from 1 insect (GER) | LC455853-LC455860 |
| | Duesseldorf | Adult | P. Mergaert | 17th July, 2017 | 100% (4/4) | - | - |
| Belgium | Brugge | Adult | P. Mergaert | 11th August, 2017 | 100% (22/22) | 8 clones from 1 insect (BEL) | LC455805-LC455812 |
| Italy | Padova | Adult; 5th | P. Mergaert | 24th August, 2017 | 100% (21/21) | 8 clones from 1 insect (ITA) | LC455829-LC455836 |
| Hungary | Szeged | Adult | P. Mergaert | 12th April, 2018 | 100% (11/11) | 8 clones from 1 insect (HUN) | LC455845-LC455852 |
| Denmark | Vejle Fjord & Billund | Adult | J. Shykoff | 20th May 2018 | 100% (4/4) | 8 clones from 1 insect (DEN) | LC455791-LC455798 |
| Ukraine | Krasiatychi | Adult | J. Shykoff | 4th June 2018 | 100% (7/7) | 8 clones from 1 insect (UKR) | LC455861-LC455868 |
| Japan | Sapporo, Hokkaido[b] | Adult | H. Itoh | 16th July, 2017 | 100% (3/3) | 8 clones from 1 insect (JPN1) | LC441114-LC441121 |
| | Sapporo, Hokkaido[b] | 5th | H. Itoh | 2nd September, 2017 | 100% (2/2) | 8 clones from 1 insect (JPN2) | LC441122-LC441129 |
| | Eniwa, Hokkaido | Adult | Y. Kikuchi | 3rd June, 2018 | 100% (5/5) | 16 clones from 2 insects (JPN3, JPN4) | LC441130-LC441145 |
| | | | | | Total: 100% (173/173) | Total: 110 clones from 14 insects | |

[a] Percent rate of *Burkholderia* infection. In parentheses, infected insects per total insects investigated are shown.

[b] Collected at the same site, but in different seasons.

**Table S2.** OTUs profile of 16S rRNA gene sequences obtained from clone library analys

| Sample ID | Analyzed clones | ***Burkholderia* (SBE group)** | | | | |
|---|---|---|---|---|---|---|
| | | **Group α** | | **Group β** | | |
| | | **OTU1** | **OTU2** | **OTU3** | **OTU4** | **OTU5** |
| FRA1 | 6 | - | 3 | 1 | - | 2 |
| FRA2 | 8 | - | - | 8 | - | - |
| FRA3 | 8 | - | - | 7 | 1 | - |
| FRA4 | 8 | - | - | 8 | - | - |
| GER | 8 | - | - | 8 | - | - |
| BEL | 8 | - | - | 8 | - | - |
| ITA | 8 | - | - | 8 | - | - |
| HUN | 8 | - | - | 8 | - | - |
| DEN | 8 | - | - | 8 | - | - |
| UKR | 8 | - | - | 8 | - | - |
| JPN1 | 8 | 7 | 1 | - | - | - |
| JPN2 | 8 | 8 | - | - | - | - |
| JPN3 | 8 | - | - | 8 | - | - |
| JPN4 | 8 | - | - | 8 | - | - |
| Total clones | 110 | 15 | 4 | 88 | 1 | 2 |

**Table S3**. Sequence identity (%) among OTUs determinded in this study.

| | OTU1 | OTU2 | OTU3 | OTU4 | OTU5 |
|---|---|---|---|---|---|
| OTU1 | - | 98.14 | 96.98 | 96.43 | 98.00 |
| OTU2 | - | - | 97.31 | 96.63 | 98.32 |
| OTU3 | - | - | - | 98.92 | 98.99 |
| OTU4 | - | - | - | - | 97.91 |
| OTU5 | - | - | - | - | - |

Accession numbers of representative sequeces of each OTU are LC441114 (OTU1), LC455799(OTU2), LC455791(OTU3), LC455818 (OTU4), and LC455800 (OTU5).
